# Differences in Risk Factors for Retinopathy of Prematurity Development in Paired Twins: A Chinese Population Study

**DOI:** 10.1155/2014/212183

**Published:** 2014-10-23

**Authors:** Gordon S. K. Yau, Jacky W. Y. Lee, Victor T. Y. Tam, Stan Yip, Edith Cheng, Catherine C. L. Liu, Benjamin C. Y. Chu, Can Y. F. Yuen

**Affiliations:** ^1^Department of Ophthalmology, Caritas Medical Centre, 111 Wing Hong Street, Kowloon, Hong Kong; ^2^Department of Applied Mathematics, The Hong Kong Polytechnic University, Kowloon, Hong Kong; ^3^Centre of Health Behaviours Research, The Chinese University of Hong Kong, Kowloon, Hong Kong

## Abstract

*Purpose.* To determine the differences in risk factors for retinopathy of prematurity (ROP) in paired twins. *Methods.* A retrospective medical record review was performed for all paired twins screened for ROP between 2007 and 2012. Screening was offered to very low birth weight (≤1500 grams) and preterm (≤32 weeks) neonates. Twins 1 and 2 were categorized based on the order of delivery. Maternal and neonatal covariates were analyzed using univariate and multivariate regression analyses for both ROP and Type 1 ROP. *Results.* In 34 pairs of Chinese twins, the mean gestational age (GA) was 30.2 ± 2.0 weeks. In Twin 1, smaller GA (OR = 0.44, *P* = 0.02), higher mean oxygen concentration (OR = 1.34, *P* = 0.03), presence of thrombocytopenia (OR = 1429.60, *P* < 0.0001), and intraventricular hemorrhage (OR = 18.67, *P* = 0.03) were significant risk factors for ROP. For Twin 2, a smaller GA (OR = 0.45, *P* = 0.03) was the only risk factor. There were no significant risk factors for ROP in Twin 1 or Twin 2 on multivariate analysis. *Conclusion.* In Chinese twin pairs, smaller GA was the only common risk factor for ROP while Twin 1 was more susceptible to the postnatal risks for ROP.

## 1. Introduction

Retinopathy of prematurity (ROP) is a vasoproliferative retinal disease targeting low birth weight, preterm neonates [1]. ROP is one of the leading causes of childhood blindness worldwide [2]. Several risk factors have been demonstrated to be associated with the development of ROP including small gestational age (GA), low birth weight (BW), anemia, blood transfusion, mechanical ventilation especially with high concentration of supplementary oxygen (FiO_2_), hypoxemia, perinatal sepsis, use of inotropes, intraventricular hemorrhage (IVH), in vitro fertilization (IVF), and multiple pregnancies [3–7].

There are only a few publications in the literature investigating the risk factors for ROP development among twin pairs [8–10]. Paired, preterm twins share the same prenatal factors and have identical gestational ages; they differ only by their birth weight and postnatal exposures. Previous studies have primarily categorized paired twins according to their BW. In a study by Woo et al. [9], it was shown that there was no significant effect of BW on ROP development or on the severity of ROP among twins, while, in Azad et al.'s [8] series, the heavier twin was found to be at greater risk for a more severe stage of ROP. The purpose of this study was to determine the differences in risk factors for ROP development in paired twins that are categorized by their order of delivery.

## 2. Patients and Methods

The study was approved by the Institutional Review Board of the Hospital Authority of Hong Kong. The study was conducted in accordance with the Declaration of Helsinki and no patient personal data was disclosed in the study. The authors declare no financial or proprietary interests.

This was a retrospective study conducted at Caritas Medical Centre, Hong Kong, which provides ophthalmological service to 2 neonatal intensive care units (NICU) for a population of 1.9 million.

Medical records for consecutive newborns of paired twins that were screened for ROP between the period of January 2007 and December 2012 were retrieved using the Clinical Data Record System of the Hospital Authority of Hong Kong.

### 2.1. ROP Screening Criterion

All preterm babies admitted to these 2 NICU with a birth weight ≤ 1500 grams and/or gestational age ≤ 32 weeks were referred to a pediatric ophthalmologist for evaluation. All eligible preterms were examined according to the screening protocol recommended by the Royal College of Ophthalmologists and United Kingdom-ROP (UK-ROP) guidelines [11, 12]. Subjects were first screened at 4 to 8 weeks of postnatal age (≥30 weeks of GA) and were examined weekly to biweekly, until retinal vascularisation reached zone 3 or feature of established ROP regression [11]. Our institution has adopted the UK screening guideline since 1996 since, in Asian countries, severe ROP can be present even in more mature and heavier babies. Treatment was diode laser implemented when the disease progressed to Type 1 ROP as per the “Early treatment for retinopathy of prematurity” (ETROP) study [13]. The staging of ROP was recorded according to the revised International Classification of ROP, including the extent, zone, and presence or absence of “plus” disease [14].

All examinations were performed by 3 experienced pediatric ophthalmologists (Gordon S. K. Yau, Victor T. Y. Tam, and Benjamin C. Y. Chu). Each infant was screened by an indirect ophthalmoscope using a 30-dioptre (D) lens after full pharmacological pupil dilatation with tropicamide 0.5% and phenylephrine 1% eye drops. A lid speculum with scleral indentation after topical anesthesia (amethocaine) was routinely used. All screening was performed under oxygen saturation monitoring and the screening was temporarily withheld in case of desaturations.

The inclusion criteria included all preterm Chinese infants of paired twins irrespective of their ultimate development of ROP or Type 1 ROP. Neonates with incomplete clinical data, non-Chinese ethnicity, and those that deceased before the completion of ROP screening were excluded.

The primary outcome measures included the severity of ROP (the extent, zone, and presence or absence of “plus” disease) as well as the 34 risk factors (both maternal and neonatal) for the development of ROP as follows.

Antenatal maternal risk factors (Tables 1 and 2):

- maternal diseases: preeclampsia (PET), gestational diabetes mellitus (GDM);
- IVF;
- use of antenatal steroid (ANS);
- preterm premature rupture of membrane.

Neonatal risk factors (Tables 1 and 2):

- demographic information (GA, BW, and gender);
- Apgar scores at 1, 5, and 10 minutes;
- postnatal interventions: surfactant administration; mechanical ventilation; use of supplementary oxygen; maintenance FiO_2_; use of nonsteroidal anti-inflammatory agents (NSAID) for patent ductus arteriosus (PDA) closure; blood transfusion; phototherapy; and total parental nutrition (TPN);
- neonatal diseases: respiratory distress syndrome (RDS); bronchopulmonary dysplasia; postnatal hypotension; congenital heart disease; PDA; anemia (defined as hemoglobin of <110 grams/litre, hematocrit < 25%); thrombocytopenia; neonatal jaundice (NNJ); IVH; necrotizing enterocolitis (NEC); hypoglycaemia; sepsis (culture positive or use of antibiotics for more than 7 days); meningitis; and acidosis based on blood Astrup results.

### 2.2. Statistics

Data were analyzed using standard univariate and multiple logistic regression analyses as implemented in the R Programming Language [15]. Most of the covariates were either binary or continuous. Estimates are also calculated independently by pregnancy order (Twin 1 or Twin 2) in order to compare the differences between their risk factors for ROP and Type 1 ROP. The responses and the nonresponses in the data were completely separated by predicators. Owing to the phenomenon of separation as described by Heinze and Schemper [16], the Penalized Maximum Likelihood Estimation method was implemented to reduce bias for related parameters as described by both Firth and Heinze et al. [17, 18]. A stepwise forward selection was used for choosing covariates for the multivariable analysis. Results were presented as odds ratios with the corresponding 95% confidence interval and *P* values. *P* value less than 0.05 was considered as statistically significant.

## 3. Results

During the study period, a total of 120 preterm infants of twin pregnancies were screened. Out of the 120 screened infants, 4 (3.3%) were of non-Chinese ethnicity, 72 infants were paired twins (60.0%), 1 infant (0.8%) did not survive before completion of ROP screening, and 1 (0.8%) had insufficient clinical information. The remaining 68 eligible infants of paired twins (34 pairs) were included for analysis.

### 3.1. Demographics

Of these 34 paired twin infants included in the study, all were of Chinese ethnicity. Eleven pairs were of different genders and 23 pairs were identical twins. Twenty-six infants (13 pairs) were the products of IVF and there was no case of twin-to-twin transfusion. Eighteen twin pairs underwent Caesarean section and 16 twin pairs had normal spontaneous deliver. There was no documented evidence of the use of instrumental delivery from the medical records. There were 39 male (57.4%) and 29 female (42.6%) subjects. The mean GA was 30.2 ± 2.0 weeks (range 25.28 to 35.0 weeks) and the mean BW was 1304.9 ± 278.6 grams (range 610.0 to 1950.0 grams). ROP of any stage developed in 9 (13.2%) infants and Type 1 ROP developed in 2 (2.9%) infants.

The mean GA of the twins was 30.2 ± 2.0 weeks (range 25.28 to 35.0 weeks). Among Twin 1's, the mean BW was 1308.9 ± 279.6 grams (range 700.0 to 1950.0 grams). ROP of any stage developed in 5 (14.7%) infants and Type 1 ROP developed in 1 (2.9%) infant belonging to Twin 1. Among Twin 2's, the mean BW was 1301.0 ± 281.8 grams (range 610.0 to 1720.0 grams). ROP of any stage developed in 4 infants (11.8%) and Type 1 ROP developed in 1 (2.9%) infant belonging to Twin 2. There was no significant difference in BW between the 2 twins (*P* = 0.4, *t*-test).

### 3.2. Risk Factors for ROP

For ROP development in Twin 1, a younger GA (OR = 0.44, *P* = 0.02), a higher FiO_2_ (OR = 1.34, *P* = 0.03), presence of thrombocytopenia (OR = 1429.60, *P* < 0.0001), and IVH (OR = 18.67, *P* = 0.03) were significant independent risk factors.

For Twin 2, a younger GA (OR = 0.45, *P* = 0.03) was the only independent risk factor for ROP development and GA was also the only consistent independent risk factor for ROP in both Twin 1 and Twin 2. There were no significant dependent risk factors for ROP development in Twin 1 or Twin 2 on multivariate analysis (Table 1).

For Type 1 ROP, there were no significant independent or dependent risk factors found in Twin 1 or Twin 2 on both univariate and multivariate regression analyses (Table 2).

**Figure 1:**
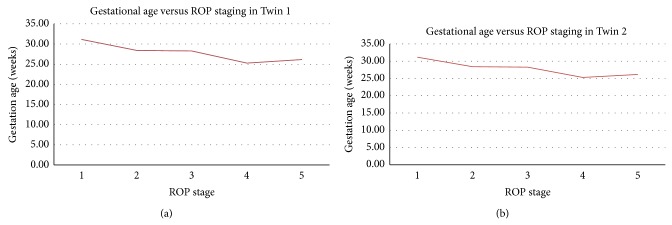
Gestational age versus ROP stage in Twins 1 and 2.

**Figure 2:**
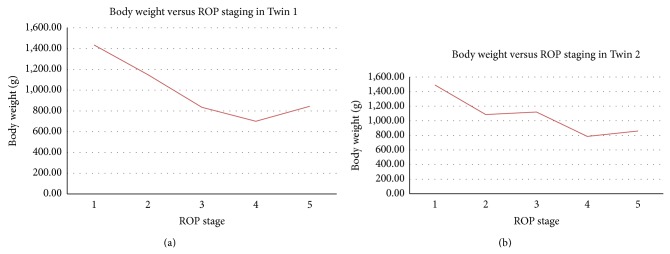
Body weight versus ROP stage in Twins 1 and 2.

The relationship between gestational age and birth weight with ROP staging has been summarized in Figures 1 and 2.

## 4. Discussion

In univariate analysis, smaller GA was the only independent risk factor for ROP development irrespective of the order of delivery while the BW was not a significant risk factor. The significance of GA as a predictor for ROP was consistent with Woo et al.'s [9] study on discordant twin pairs where infants with GA ≤ 28 weeks were associated with significantly (*P* < 0.05) higher risk of ROP development, stage 3 ROP, and threshold ROP requiring laser treatment (76.5%, 52.9%, and 29.4%, resp.) compared to those with GA > 28 weeks (15.8%, 0.0%, and 0.0%, resp.). On the other hand, in a twin pair study by Azad et al. [8], it was shown that there were no significant differences in both GA and BW (*P* = 1.00 for GA, *P* = 0.39 for BW) among the twins with severe ROP (mean GA of 29.3 ± 1.3 weeks and mean BW of 1239 ± 206 grams) compared to those with less severe ROP (mean GA of 29.3 ± 1.3 weeks and mean BW of 1297 ± 188 grams).

Unlike in previous studies that categorized paired twins based on ROP severity or BW, we categorized the twins based on their order of delivery which has not been reported before in the literature. We feel that this classification is both clinically relevant for clinicians during their management of the twins and important for parents who may have questions about the risk of ROP in their twins. In our study, there were discrepancies in the susceptibility of postnatal risk factors for ROP between the twins despite exposure to similar postnatal conditions. Twin 1 was more likely to develop ROP when exposed to the following factors that were found to be independent ROP risk factors in Twin 1 but not Twin 2: presence of IVH, thrombocytopenia, and higher FiO_2_. These findings suggest that the first delivered infant of twin pregnancies may be more prone to develop ROP when exposed to the above risk factors as compared to the second infant. This prompts clinicians to be even more vigilant when screening for ROP in the first delivered twin, especially in the presence of the above risk factors.

A previous study by Prins [19] investigating the postnatal comorbidities among twins found that the second delivered infant was associated with higher risks of postnatal complications including intubation and RDS when compared to their twin. The author postulated that this observation was partly attributed by the smaller birth weight of the second twin rather than due to the order of delivery. Wadhawan et al. [20], on the other hand, found that there was no difference in neurodevelopmental impairment among twin pairs regardless of the order of delivery. In our study, there was no significant difference in BW between Twins 1 and 2 (*P* = 0.4); thus, the observed differences in ROP risk factors among the twins were most probably not related to BW. Our findings suggest that, in contrast to previous beliefs, Twin 2 was not necessarily the weaker twin and that Twin 1 should receive an equal amount if not a more vigilant monitoring and control for the risk factors leading to ROP. Larger scale prospective cotwin pair studies are warranted to provide an exact pathophysiological explanation to the differences in susceptibility to ROP among the twins.

To the best of our knowledge, this is the first study reporting the incidence and risk factors of ROP and Type 1 ROP in paired twins of Chinese infants based on the order of delivery. This study serves as a platform for future prospective and multicenter studies in this area as we anticipate that the number of preterm twins will likely continue to grow as a result of the growing demands of assisted reproduction.

Our study had its limitations. Firstly, the retrospective nature of this study inevitably generates inconsistencies in data although every effort was made to exclude subjects with incomplete clinical data. Secondly, subjects were screened by 3 pediatric ophthalmologists at different time points and minor interobserver variability can exits, which has not been objectively accounted for in this study but as all were trained to follow a strict ROP screening guideline and given the large population requiring screening, it was the most optimal balance in terms of providing clinical service and standardization for research. Lastly, there were only a small number of infants included in our study due to the rarity of preterm twins; with over 34 risk factors and only 34 pairs of twins, the distribution of risk factors among twin pairs were likely to be uneven and hence negative risk factors should be interpreted with care taking into account this limitation. Nevertheless, this study provides important data on the incidence and risk factors of ROP in the preterm Chinese infants from paired twins using more updated ROP screening guidelines than what currently exists in the literature.

## 5. Conclusion

In preterm, Chinese paired twins, a smaller GA was the only common independent risk factor for the development of ROP. The twin that was delivered first was more susceptible than the other twin to ROP development if they had IVH, thrombocytopenia, or higher FiO_2_ exposure.

## Figures and Tables

**Table 1:** Univariate and multivariate analysis of maternal and natal covariates for ROP development in twin pairs.

	Univariate logistic analysis						Multivariate logistic analysis					
	Twin 1			Twin 2			Twin 1			Twin 2		
	Odds ratio	Coefficient estimates	*P* value	Odds ratio	Coefficient estimates	*P* value	Odds ratio	Coefficient estimates	*P* value	Odds ratio	Coefficient estimates	*P* value
Gender (male versus female)	1.22	0.20	0.84	0.67	−0.41	0.70	0.99	−0.01	0.71	1.00	0.00	0.92
Gestational age	0.44	−0.82	0.02∗	0.45	−0.79	0.03∗	1.00	0.00	1.00	1.00	0.00	1.00
Birth weight	1.00	0.00	0.10	1.00	0.00	0.09	1.00	0.00	1.00	1.00	0.00	1.00
Preeclampsia	0.01	−4.29	0.29	0.02	−3.93	1.00	1.00	0.00	0.98	1.00	0.00	1.00
Gestational diabetes mellitus	2.17	0.77	0.54	1.67	0.51	0.68	1.00	0.00	0.97	1.00	0.00	1.00
In vitro fertilization	1.09	0.09	0.93	2.00	0.69	0.52	1.00	0.00	0.96	1.00	0.00	1.00
Postnatal hypotension	3.37	1.22	0.36	0.02	−3.93	1.00	1.00	0.00	1.00	1.00	0.00	0.99
Inotropes use	7.00	1.95	0.20	9.67	2.27	0.14	1.00	0.00	0.99	1.00	0.00	1.00
Antenatal steroid use	76.10	4.33	1.00	50.51	3.92	1.00	1.00	0.00	1.00	1.00	0.00	1.00
Apgar score 1 min	0.75	−0.28	0.24	1.00	0.00	0.99	1.00	0.00	0.99	1.00	0.00	1.00
Apgar score 5 min	0.71	−0.34	0.30	1.16	0.15	0.75	1.00	0.00	0.96	1.00	0.00	1.00
Apgar score 10 min	0.51	−0.67	0.13	2.09	0.74	0.48	1.00	0.00	1.00	1.00	0.00	1.00
Respiratory distress syndrome	85.99	4.45	1.00	53.82	3.99	1.00	1.00	0.00	0.75	1.00	0.00	0.98
Bronchopulmonary dysplasia	2.10	0.74	0.46	1.33	0.29	0.82	1.00	0.00	1.00	1.00	0.00	1.00
Surfactant use	85.99	4.45	1.00	53.82	3.99	1.00	1.00	0.00	0.75	1.00	0.00	0.98
Invasive mechanical ventilation	4.71	1.55	0.13	2.33	0.85	0.43	1.00	0.00	0.95	1.00	0.00	1.00
Oxygen supplement	0.14	−1.95	0.20	50.51	3.92	1.00	1.00	0.00	1.00	1.00	0.00	1.00
Mean oxygen concentration	1.34	0.29	0.03∗	0.95	−0.05	0.72	1.00	0.00	0.99	1.00	0.00	0.99
Congenital heart disease	1.56	0.45	0.72	2.00	0.69	0.52	1.00	0.00	0.99	1.00	0.00	0.99
Patent ductus arteriosus	5.75	1.75	0.09	3.92	1.37	0.26	1.00	0.00	1.00	1.00	0.00	1.00
NSAID use	5.78	1.75	0.11	0.92	−0.09	0.94	1.00	0.00	1.00	1.00	0.00	1.00
Anemia	177.43	5.18	1.00	0.78	−0.25	0.84	1.00	0.00	0.98	1.00	0.00	1.00
Thrombocytopenia	1429.60	7.27	<0.0001∗	0.02	−3.93	1.00	1.00	0.00	1.00	1.00	0.00	1.00
Blood transfusion	10.50	2.35	0.05	0.92	−0.09	0.94	1.00	0.00	0.99	1.00	0.00	1.00
Intraventricular hemorrhage	18.67	2.93	0.03∗	0.02	−3.99	0.26	1.00	0.00	0.99	1.00	0.00	1.00
Necrotizing colitis	0.01	−4.31	0.09	9.67	2.27	0.14	1.00	0.00	1.00	1.00	0.00	1.00
Neonatal jaundice	1.27	0.24	0.84	0.25	−1.39	0.21	1.00	0.00	1.00	1.00	0.00	1.00
Phototherapy	1.52	0.42	0.72	0.30	−1.19	0.27	1.00	0.00	1.00	1.00	0.00	1.00
Total parenteral nutrition	2.10	0.74	0.46	1.33	0.29	0.82	1.00	0.00	0.99	1.00	0.00	1.00
Hypoglycemia	0.83	−0.18	0.88	50.51	3.92	1.00	1.00	0.00	1.00	1.00	0.00	1.00
Sepsis	0.96	−0.04	0.97	0.78	−0.25	0.84	1.00	0.00	1.00	1.00	0.00	1.00
Meningitis	0.56	−0.59	0.62	2.17	0.77	0.54	1.00	0.00	1.00	1.00	0.00	0.98
Preterm premature rupture of membrane	0.41	−0.89	0.45	2.00	0.69	0.52	1.00	0.00	1.00	1.00	0.00	1.00
Acidosis	0.71	−0.34	0.73	1.33	0.29	0.82	1.00	0.00	1.00	1.00	0.00	1.00

^*^Statistically significant.

**Table 2:** Univariate and multivariate analysis of maternal and natal covariates for Type 1 ROP development in twin pairs.

	Univariate logistic analysis						Multivariate logistic analysis					
	Twin 1			Twin 2			Twin 1			Twin 2		
	Odds ratio	Coefficient estimates	*P* value	Odds ratio	Coefficient estimates	*P* value	Odds ratio	Coefficient estimates	*P* value	Odds ratio	Coefficient estimates	*P* value
Gender (male versus female )	31.51	3.45	1.00	0.07	−2.64	1.00	1.01	0.01	1.00	1.00	0.00	1.00
Gestational age	0.26	−1.33	0.16	0.70	−0.36	1.00	1.01	0.01	1.00	0.99	−0.01	1.00
Birth weight	1.00	0.00	0.27	0.99	−0.01	0.17	1.00	0.00	1.00	1.00	0.00	1.00
Preeclampsia	0.04	−3.15	1.00	0.04	−3.15	1.00	1.00	0.00	1.00	1.00	0.00	0.98
Gestational diabetes mellitus	0.05	−2.95	1.00	44.08	3.79	1.00	1.00	0.00	1.00	1.00	0.00	0.97
In vitro fertilization	19.39	2.96	1.00	25.34	3.23	1.00	1.00	0.00	0.99	1.00	0.00	0.96
Postnatal hypotension	0.04	−3.15	1.00	0.04	−3.15	1.00	1.00	0.00	0.99	1.00	0.00	1.00
Inotrope use	0.03	−3.51	1.00	314.19	5.75	1.00	1.00	0.00	0.99	1.00	0.00	0.99
Antenatal steroid use	17.29	2.85	1.00	19.16	2.95	1.00	1.00	0.00	1.00	1.00	0.00	0.99
Apgar score 1 min	0.29	−1.24	0.20	0.84	−0.17	0.71	1.00	0.00	1.00	1.00	0.00	0.97
Apgar score 5 min	0.49	−0.71	0.23	0.42	−0.86	0.19	1.00	0.00	0.98	1.00	0.00	0.96
Apgar score 10 min	0.68	−0.38	0.64	0.36	−1.02	0.44	1.00	0.00	0.97	1.00	0.00	0.94
Respiratory distress syndrome	17.04	2.84	1.00	16.62	2.81	1.00	1.00	0.00	1.00	1.00	0.00	1.00
Bronchopulmonary dysplasia	0.06	−2.87	1.00	58.74	4.07	1.00	1.00	0.00	1.00	1.00	0.00	1.00
Surfactant use	17.04	2.84	1.00	16.62	2.81	1.00	1.00	0.00	1.00	1.00	0.00	1.00
Invasive mechanical ventilation	45.38	3.82	1.00	33.64	3.52	1.00	1.00	0.00	1.00	1.00	0.00	0.98
Oxygen supplement	33.56	3.51	1.00	19.16	2.95	1.00	1.00	0.00	1.00	1.00	0.00	0.98
Mean oxygen concentration	1.39	0.33	0.18	0.86	−0.15	0.62	1.00	0.00	1.00	1.00	0.00	1.00
Congenital heart disease	63.09	4.14	1.00	25.34	3.23	1.00	1.00	0.00	1.00	1.00	0.00	1.00
Patent ductus arteriosus	61.68	4.12	1.00	9.51	2.25	1.00	1.00	0.00	1.00	1.00	0.00	0.99
NSAID use	0.06	−2.85	1.00	61.68	4.12	1.00	1.00	0.00	1.00	1.00	0.00	0.99
Anemia	15.06	2.71	1.00	45.38	3.82	1.00	1.00	0.00	1.00	1.00	0.00	1.00
Thrombocytopenia	0.01	−4.98	1.00	0.04	−3.15	1.00	1.00	0.00	0.99	1.00	0.00	0.99
Blood transfusion	25.34	3.23	1.00	61.68	4.12	1.00	1.00	0.00	1.00	1.00	0.00	0.99
Intraventricular hemorrhage	161.31	5.08	1.00	0.03	−3.51	1.00	1.00	0.00	1.00	1.00	0.00	1.00
Necrotizing colitis	0.03	−3.51	1.00	0.03	−3.51	1.00	1.00	0.00	0.99	1.00	0.00	0.98
Neonatal jaundice	17.04	2.84	1.00	0.03	−3.52	1.00	1.00	0.00	1.00	1.00	0.00	1.00
Phototherapy	17.84	2.88	1.00	0.02	−3.98	1.00	1.00	0.00	1.00	1.00	0.00	1.00
Total parenteral nutrition	0.06	−2.87	1.00	58.74	4.07	1.00	1.00	0.00	1.00	1.00	0.00	1.00
Hypoglycemia	16.62	2.81	1.00	19.16	2.95	1.00	1.00	0.00	1.00	1.00	0.00	0.99
Sepsis	0.06	−2.81	1.00	45.38	3.82	1.00	1.00	0.00	1.00	1.00	0.00	1.00
Meningitis	45.38	3.82	1.00	0.06	−2.85	1.00	1.00	0.00	1.00	1.00	0.00	1.00
Preterm premature rupture of membrane	0.05	−3.09	1.00	25.34	3.23	1.00	1.00	0.00	1.00	1.00	0.00	1.00
Acidosis	0.03	−3.52	1.00	0.06	−2.81	1.00	1.00	0.00	1.00	1.00	0.00	1.00
